# What Antarctic Plants Can Tell Us about Climate Changes: Temperature as a Driver for Metabolic Reprogramming

**DOI:** 10.3390/biom11081094

**Published:** 2021-07-23

**Authors:** Laura Bertini, Flora Cozzolino, Silvia Proietti, Gaia Salvatore Falconieri, Ilaria Iacobucci, Rosanna Salvia, Patrizia Falabella, Maria Monti, Carla Caruso

**Affiliations:** 1 Department of Ecological and Biological Sciences, University of Tuscia, 01100 Viterbo, Italy; lbertini@unitus.it (L.B.); s.proietti@unitus.it (S.P.); gfalconieri@unitus.it (G.S.F.); 2 Department of Chemical Sciences, University of Naples Federico II, 80126 Naples, Italy; flora.cozzolino@unina.it (F.C.); iacobucci@ceinge.unina.it (I.I.); 3 CEINGE Advanced Biotechnologies, University of Naples Federico II, 80145 Naples, Italy; 4 Department of Sciences, University of Basilicata, 85100 Potenza, Italy; r.salvia@unibas.it (R.S.); patrizia.falabella@unibas.it (P.F.); 5 Spinoff XFlies s.r.l, University of Basilicata, 85100 Potenza, Italy

**Keywords:** *Colobanthus quitensis*, differential proteomic analysis, open top chambers, response to stress, temperature changes

## Abstract

Global warming is strongly affecting the maritime Antarctica climate and the consequent melting of perennial snow and ice covers resulted in increased colonization by plants. *Colobanthus quitensis* is a vascular plant highly adapted to the harsh environmental conditions of Antarctic Peninsula and understanding how the plant is responding to global warming is a new challenging target for modern cell physiology. To this aim, we performed differential proteomic analysis on *C. quitensis* plants grown in natural conditions compared to plants grown for one year inside open top chambers (OTCs) which determine an increase of about 4 °C at midday, mimicking the effect of global warming. A thorough analysis of the up- and downregulated proteins highlighted an extensive metabolism reprogramming leading to enhanced photoprotection and oxidative stress control as well as reduced content of cell wall components. Overall, OTCs growth seems to be advantageous for *C. quitensis* plants which could benefit from a better CO_2_ diffusion into the mesophyll and a reduced ROS-mediated photodamage.

## 1. Introduction

Antarctica is one of the last pristine environments where endemic organisms have progressively specialized to deal with very harsh weather conditions, living often at their physiological limit. This specialization makes them extremely susceptible to any minimal environmental change that could be responsible for the extinction of some species. The Antarctic Peninsula is one of the areas on Earth mostly affected by global warming which is inducing a temperature rise at an alarming rate. In the past 50 years, the mean annual temperature has risen by approximately 2.6 °C and the mean summer air temperature by 1.5 °C along the west coast of the Antarctic Peninsula [1,2]. This makes maritime Antarctic an open-air laboratory where the study of the genetic and molecular traits which drive the adaptation of living organisms to rapidly changing environmental conditions may permit the disclosure of molecular biomarkers for efficient climate change monitoring. The climatic conditions of this ecosystem allowed the colonization and survival of only two endemic vascular plants: the pearlwort *Colobanthus quitensis* (Kunth) Bartl. (Caryophyllaceae) and the hairgrass *Deschampsia antarctica* Desv. (Poaceae) [3]. Both of them display many morphological and physiological traits related to resistance against constantly low temperatures and other environmental stressors such as drought, low nutrient availability, and high irradiance. In particular, Antarctic plants exhibit anatomical adaptations typical of xerophytes such as small and thick leaves and lignified cell walls, associated with thick and tight leaf mesophyll which limits CO_2_ conductance [4]. Moreover, they evolved many other physiological adaptations such as freezing tolerance, ability to maintain positive photosynthetic rate near 0 °C, resistance to photoinhibitory conditions, and tolerance to water stress ([5] and references therein). Furthermore, a large number of studies are focusing on the role of biotic interactions in plant adaptation and fitness, exploring the leaf-associated microbiota [6,7,8]. The registered warmer temperature allowed spreading of both plant species due to longer growing seasons, glacier retreats, and an increase in precipitation and plant nutrient availability [9,10]. These environmental changes were associated with increased growth and reproduction rates as well as greater production of viable seeds of both species [11]. Moreover, the photosynthetic rate was also positively influenced by small temperature increases, mainly in *C. quitensis*, improving the vegetative growth [12]. However, it is important to point out that Antarctic plants have developed mechanisms of resistance to low temperatures during millions of years of evolution, and the resilience they have developed in their natural environment could slow down the response to future climate change. On the other hand, warming can reduce plant freezing survival [13] impairing their resistance if the tendency to warm up ceases. Actually, Turner et al. [14] reported that warming in the Antarctic Peninsula stopped during recent decades, even if new warming episodes are likely to occur during this century. Under this natural climate variability, the study of the ecophysiological traits of Antarctic vascular plants that drive their adaptation to cold and warmer growth conditions is a new target for modern plant physiology. Indeed, many papers have recently focused on the analysis of climate influence on anatomical and photosynthetic parameters ([12] and references therein).

Recent developments in “omics” disciplines have opened up new perspectives towards a comprehensive understanding of biological processes related to plant stress responses. In particular, proteomics offers a snapshot of cell metabolism taking into account only the functional molecules that actively contribute to plant cell viability. In a recent work, Clemente–Moreno and colleagues [15] combined metabolomics, ecophysiology, biochemistry, and statistical modeling approaches to understand the mechanisms leading to low temperature tolerance in two *C. quitensis* ecotypes revealing that besides anatomical structures, long-term stress tolerance mechanisms in *C. quitensis* seem to be sustained by sulfur-based molecules, polyamines, and secondary metabolites.

In order to deepen knowledge on the mechanisms underlying *C. quitensis* adaptation to climate change, we performed the de novo assembly of *C. quitensis* transcriptome of plants grown in natural condition (OUT samples) versus plants grown for one year inside small greenhouses open on the top, namely open top chambers (OTC samples), which determine an increase of about 4 °C during midday, mimicking the effect of global warming [16]. Indeed, OTCs are widely recognized as a useful tool for simulating a warmer climate and studying the response of high-latitude ecosystems to warming [17,18,19].

In this work, we extended the information previously obtained through transcriptomic investigations by adopting a differential proteomic approach. Analyses were performed on OTC versus OUT samples in order to provide a global view of proteome reprogramming triggered by different growth conditions. To combine transcriptomic and proteomic data we built a custom home-made *C. quitensis* protein database reporting the six possible putative protein sequences of each *C. quitensis* transcript. This approach is now widely used because it allows the reliable identification of really expressed proteins. Furthermore, this approach is particularly useful for the identification of proteins from organisms whose sequences are not available in public databases. The proteome profiling has been analyzed in-depth by using a combination of high throughput profiling techniques and bioinformatics tools. Moreover, gene ontology (GO) analysis has been carried out to obtain more comprehensive insight into the biological processes affected by temperature. Interestingly, we found that plants grown at warmer temperature are more protected from photooxidative damage, thus suffering less from photoinhibition. Moreover, proteins supporting photorespiration efficiency were found over-expressed, suggesting that RUBISCO oxygenase activity could prevent photoinhibition by reducing ROS-mediated photodamage. Moreover, redox homeostasis appeared to be also supported by the over-expression of some antioxidant enzymes and temperature-induced proteins known to be involved in maintaining the redox state. As for the metabolic processes, higher growth temperatures were found to reduce the synthesis of complex carbohydrates and proteins, being cellular catabolic processes generally upregulated.

## 2. Materials and Methods

### 2.1. Sample Collection

Field activity was carried out in close proximity of the Henryk Arctowski Antarctic Research station, King George Island, Maritime Antarctica (62°14′ S, 58°48′ W). Samples were collected inside the Antarctic Specially Protected Area (ASPA) 128 using permits provided by The Chilean Antarctic Institute (INACH) and by the Italian National Agency for New Technologies, Energy and Sustainable Economic Development—Technical Antarctic Unit (ENEA-UTA). *Colobanthus quitensis* plants were grown in the field inside OTC from December 2012 to March 2014. The OTC were similar to those used in the International Tundra Experiment (ITEX) and, in particular, they were made with transparent Plexiglass^®^ walls of 40 cm height, punched with 25 holes of 1.5 cm diameter each to allow some wind to pass through. Temperature sensors inside OTC revealed an average increase of 4 °C in the internal air temperature during midday [13]. Other parameters, such as mean PAR and relative humidity, were not altered by OTC [12]. Control plots were selected approximately 2–5 m away from each OTC, in random direction. Leaf samples from individuals grown either inside OTC (OTC samples) or in natural conditions (OUT samples) were harvested and pooled. Freshly collected leaves were soaked in RNAlater^®^ solution (2:10, *w/v*) (Sigma–Aldrich, Saint Louis, MO, USA) to preserve RNA and proteins from degradation, and stored at −20 °C during Antarctic stay. The samples were first moved to Chile and then to Italy maintaining the cold chain. Once in Italy, they were stored at −80 °C until RNA and protein extraction.

### 2.2. Colobanthus Quitensis Protein Database Building

The previously assembled and annotated *Colobanthus quitensis* transcriptome [16] was used to generate a custom-made protein database. Starting from the 165,508 contigs obtained from the transcriptomic analysis, the six reading frames were translated in their corresponding amino acid sequences by SEQtools software (http://www.seqtools.dk/, accessed on 15 December 2017), thus obtaining 993,048 predicted amino acid sequences (“*C. quitensis* protein database”). This database provides useful information for proteins identification allowing the combination of transcriptomic and proteomic data, as described in Results and Discussion section.

### 2.3. Protein Extraction for Proteomic Analysis

Two biological replicates for both OTC and OUT samples were finely ground using a mortar and pestle under continuous addition of liquid nitrogen and then a protein extraction buffer (4% SDS, 20 mM Tris-HCl pH 8.0, 1 mM phenylmethylsulfonyl fluoride (PMSF), protease inhibitor cocktail) was added. Approximately 300 µL of buffer was added to each sample, and afterwards incubated for 30 min at 4 °C on a stirring wheel and then centrifuged at 13,000 rpm for 30 min. After centrifugation, supernatants containing the protein fraction were collected and protein concentration was determined by the Bradford assay (Bio-rad, Hercules, CA, USA) [20].

### 2.4. SDS-PAGE and Mass Spectrometry Analysis

Fifty micrograms of protein extracts from two biological replicates of both OTC and OUT samples were diluted in Laemmli buffer [21] containing 100 mM dithiothreitol (DTT), boiled for 10 min, and loaded onto a 10% bis-acrylamide gel. After SDS-PAGE separation, the gel was stained with Coomassie Brilliant Blue, and 19 slices were cut from each lane. Each slice was subjected to in situ digestion as previously reported [22]. Finally, the obtained peptide mixtures were collected, vacuum-dried, and then resuspended in 0.2% formic acid for LC-MS/MS analyses. LC-MS/MS analyses were carried out onto an LTQ Orbitrap XL (ThermoScientific, Waltham, MA, USA) coupled with a nanoLC system (nanoEasy II). Peptide mixtures were separated onto a C18 capillary column (200 mm, 75 µm ID, 5 µm, 120 Å, Nanoseparation, NL) by using a linear gradient of eluent B (0.2% formic acid in 95% acetonitrile LC-MS Grade) from 5% to 95% in 87 min at a flow rate of 250 nL/min. The mass spectrometry analyses were performed using the data-dependent acquisition (DDA) mode: From each MS scan in the range from 400 to 1800 *m*/*z*, the ten most abundant ions were selected and fragmented in collision-induced dissociation (CID) conditions, applying a dynamic exclusion window of 40 s. Each sample was run in duplicates.

### 2.5. Protein Identification and Quantification and Statistical Analyses

LC-MS/MS raw data were processed by MaxQuant 1.5.2 integrated with Andromeda search engine, and the customized “*Colobanthus quitensis* protein database” was used for protein identification. The parameters employed for protein identification were: minimum 4 peptides, at least 2 unique; methionine oxidation and pyroglutamate formation on N-terminal glutamine as variable modifications; 20 ppm was the accuracy for the first search, then lowered to 4.5 ppm in the main search; and 0.01 FDR with a reverse database for decoy was used. Protein quantification was carried out for proteins showing minimum 2 unique and razor peptides identified. The only modification allowed for quantification was the carbamidomethylation of Cys residues. Protein relative quantification was obtained by using a spectral counts (SpCs) based method. In details, SpCs from MaxQuant were normalized by using RSC method [23], and the means were calculated for technical duplicates. The obtained values were introduced in MultiExperiment Viewer (MeV), performing the unpaired Student’s *t*-test imposing a cutoff *p*-value < 0.01 for the statistical significance. The protein normalized spectral counts in both conditions (OTC and OUT) were used to calculate the fold change (FC) as log_2_ OTC/OUT. The volcano plot was computed by VolcaNoseR [24] with cutoff values spanning from −0.5 to 0.4 for log_2_FC and 2 for −log_10_ *p*-value.

### 2.6. Bioinformatic Analysis

Gene ontology (GO) enrichment analysis was conducted using singular enrichment analysis (SEA) in AgriGO toolkit version 1.2 (http://bioinfo.cau.edu.cn/agriGO/analysis.php, accessed on 15 May 2019) [25]. The Arabidopsis Information Resource (TAIR) code of the orthologous *Arabidopsis thaliana* proteins was used as input and the TAIR10 genome assembly as background. Statistical analysis included Fisher’s test and the Yekutieli multiple-test with a threshold of FDR = 0.05.

Protein–protein interaction (PPI) networks were analyzed using the STRING program version 11.0 (http://string-db.org/, accessed on 3 June 2019) [26] which predicts PPI employing a mixture of prediction approaches and a combination of experimental data (neighborhood, gene fusion, co-expression, experiments, databases, text mining, co-occurrence). Networks were performed at 0.7 confidence level.

### 2.7. Enzymatic Activity Assays

One gram of *C. quitensis* leaves (fresh weight) was finely ground with a mortar and pestle under continuous addition of liquid nitrogen. The powder was resuspended in 5 mL of 50 mM of cold sodium phosphate buffer (pH 7.6) containing 1 mM EDTA, 4% (*w/v*) polyvinylpyrrolidone, 3 mM DTT, and a cocktail of protease inhibitors (Complete ULTRA tablets, Roche). After centrifugation at 9000 rpm for 15 min at 4 °C, the protein content was evaluated according to the method described by Bradford [20] and the supernatant was used for enzyme activity assays as already described [27] with minimal changes. Briefly, catalase (CAT) activity was measured monitoring the absorbance decrease at 240 nm caused by the decomposition of hydrogen peroxide (ε = 0.0436 mM^−1^ cm^−1^). The reaction mixture contained 19 mM of H_2_O_2_ in 50 mM of potassium phosphate buffer (pH 7.6). Ascorbate peroxidase (APX) activity was assayed by estimating the absorbance decrease at 290 nm due to the ascorbate oxidation (ε = 2.8 mM^−1^ cm^−1^). The reaction mixture contained 0.1 mM of H_2_O_2_, 0.5 mM of ascorbate, and 0.1 mM of EDTA in 50 mM of potassium phosphate buffer (pH 7.6). Guaiacol peroxidase (POD) activity was measured monitoring the absorbance increase at 470 nm caused by the reduction of guaiacol to form tetraguaiacol (ε = 26.6 mM^−1^ cm^−1^). The reaction mixture contained 0.4% guaiacol (*v/v*) and 0.03% H_2_O_2_ in 100 mM potassium phosphate buffer (pH 7.6). Glutathione-S transferase (GST) activity was determined by measuring the absorbance increase at 340 nm caused by the conjugation of 1-chloro-2,4-dinitrobenzene (CDNB) (ε = 9.6 mM^−1^ cm^−1^) with reduced glutathione (GSH). The reaction mixture contained 1 mM of CDNB, 1 mM of GSH in 100 mM of potassium phosphate buffer (pH 6.5) containing 1 mM of EDTA. All the above enzyme activities were expressed as unit mg^−1^ protein. Superoxide dismutase (SOD) activity was assayed using the SOD determination kit (Sigma–Aldrich, Uppsala, Sweden) following the manufacturer’s instructions. The kit is based on the use of Dojindo’s highly water-soluble tetrazolium salt, WST-1 (2-(4-iodophenyl)-3-(4-nitrophenyl)-5-(2,4-disulfophenyl)-2H-tetrazolium, monosodium salt), which produces a water-soluble formazan dye upon reduction by a superoxide anion with a maximum of absorbance at 440 nm. The rate of the reduction is linearly related to the xanthine oxidase activity which generates the superoxide anion, and is inhibited by SOD. Thus, the SOD activity is quantified as an inhibition activity (IC_50_) by measuring the decrease of absorbance at 440 nm.

### 2.8. Thiobarbituric Acid Reactive Substance Measurement

The level of thiobarbituric acid reactive substances (TBARS) was measured to assess lipid peroxidation following the protocol already described [27]. Briefly, 400 mg of *C. quitensis* leaves (fresh weight) were finely ground using a mortar and pestle under continuous addition of liquid nitrogen. The powder was resuspended in 3 mL of trichloroacetic acid (TCA) 0.1% and mixed on the vortex. Following centrifugation at 13,500 rpm for 10 min, 400 μL of the supernatant (or 400 μL of 0.1% TCA for the blank) were added either to 1 mL of 0.5% TBA in 20% trichloroacetic acid (+TBA solution) or to 1 mL of 20% trichloroacetic acid (–TBA solution) (dilution factor 1:3.5). Samples were incubated at 80 °C for 30 min and then cooled on ice. After centrifugation at 13,500 rpm for 5 min, the absorbance was measured both at 532 nm, which represents the maximum absorbance of the TBA-TBARS complex, and 600 nm to allow correction for nonspecific turbidity. To calculate the TBARS equivalent (nmol mL^−1^), the ε_μM_ of malondialdehyde (MDA), one of the main products of membrane damage, was used according to the following formula:

[A/ε_μM_ MDA (0.155 μM^−1^ cm^−1^)] × dilution factor

where A = [(A_532nm (+TBA)_ − A_600nm (+TBA)_) − (A_532nm (−TBA)_ − A_600nm (−TBA)_)]

### 2.9. RNA Extraction and Quantitative Reverse Transcriptase–PCR Analysis

Total RNA was extracted using the Nucleospin^®^ RNAPlant kit (Macherey–Nagel, Düren, Germany), starting from 100 mg of finely ground leaf samples, in accordance with the manufacturer’s instructions. RNA concentration was estimated by reading spectrophotometric absorbance at 260 nm, whereas the OD_260_/OD_280_ and OD_260_/OD_230_ nm absorption ratios were calculated to evaluate RNA quality and purity (spectrophotometer UV-30 SCAN, ONDA). RNA integrity was also verified by agarose gel electrophoresis, whereas the absence of DNA contamination was tested using 100 ng of total RNA as a template in a PCR reaction using *EF1α* specific primers for amplification. Complementary DNA (cDNA) was synthesized using the ImProm-II™ reverse transcription system (Promega, Madison, WI, USA) starting from 1 μg of RNA as template and using the oligo-dT primer for first strand synthesis.

Sequence information of the genes to be amplified were obtained from the shotgun transcriptome assembly of *C. quitensis* leaves (National Center of Biotechnology Information (NCBI) Sequence Read Archive (SRA), accession SRX814890) [16]. For primer pairs design, we searched for all the contigs coding for each of the selected genes inside the transcriptome data and we aligned them using the freely available ClustalW bioinformatics tool (https://embnet.vital-it.ch/software/ClustalW.html, accessed on 21 March 2016). Then, primers were designed inside the most conserved region using Primer3 software (http://bioinfo.ut.ee/primer3-0.4.0/, accessed on 28 March 2016). The specificity of each selected primer pair was observed via standard PCR on synthesized cDNA using the BIOTAQ DNA polymerase (Bioline, London, UK), and each amplification product was verified by 1.5% agarose gel electrophoresis. All primers used in the present study are listed in Table S1.

Quantitative reverse transcriptase—PCR (qRT-PCR) reactions were performed in 96-well plates on a Bio-Rad CFX96 real-time PCR thermal cycler (Bio-Rad, Hercules, CA, USA), using the SYBR green detection system. The reaction mixture (10 μL) contained 1 μL of four-fold diluted cDNA, 5 μL of Sso Advanced SYBR Green Supermix (Bio-Rad, Hercules, CA, USA), and a different concentration of each gene specific primer (Table S1). The cycling conditions were the following: initial denaturation step at 95 °C for 3 min, followed by 44 cycles at 95 °C for 10 s, and primer specific annealing temperature for 30 s (Table S1). Following amplification, the melting curves ranging from 70 to 95 °C (with a constant increase of 0.5 °C every 5 s) were evaluated in order to check the PCR specificity (Figure S1). Each assay included no-template controls (NTCs) for each primer pair. Primer efficiency (*E*) was calculated by generating standard curves for each oligonucleotide pair with at least five serial fourfold dilution points and the slope of the amplification curve was used to calculate *E* = 10^(−1/slope)^. The gene expression of selected protein-coding genes was normalized against the reference genes glucose 6-phosphate dehydrogenase (*G6PDH*) and catalytic subunit of protein phosphatase 2A (*PP2Acs*), as suggested in [28]. The relative gene expression was determined using the 2^−ΔΔCt^ method. Output data were processed using the CFX Manager^TM^ Software (Bio-Rad, Hercules, CA, USA). All qRT-PCR reactions were run in three technical and biological replicates.

### 2.10. Data Analyses

For enzymatic activity assays and qRT-PCR analysis the differences between the two growth conditions were compared applying the Student’s *t*-test.

Results were expressed as means ± standard deviation (SD) of three biological and three technical replicates and *p* < 0.05 was considered as statistically significant.

## 3. Results and Discussion

**Figure 1:**
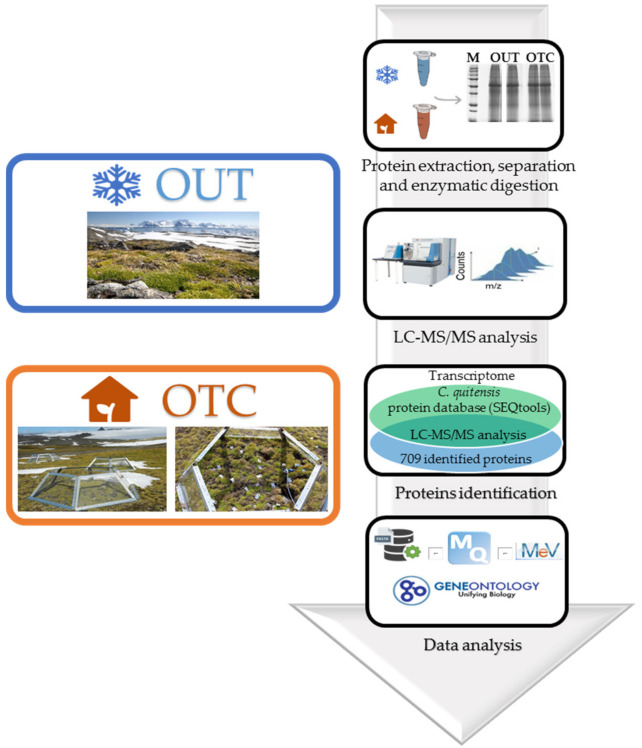
Experimental workflow. The open top chambers used in the experiment are shown.

Global warming is one of the major threats all over the world and the Antarctic Peninsula is mainly affected by this stressor. The temperature rise influences the life of endemic organisms and the study of its effect on proteome reprogramming of local plants may open up new perspectives on their adaptation to environmental changes. In this work, a differential proteomic analysis was carried out between *C. quitensis* plants grown in natural conditions (OUT samples) and inside open top chambers (OTC samples) which allow plants to live at slightly warmer temperature, mimicking the effect of global warming. An overview of the experimental proteomic workflow is shown in Figure 1.

### 3.1. De Novo Colobanthus Quitensis Protein Database Building

The integrated transcriptomic and proteomic approach is the most reliable and widely used strategy [29,30,31] for the identification of actually expressed proteins, especially with respect to strategies based exclusively on transcriptomic analyses that allow only the identification of putative proteins without verifying their real expression within the cells. Furthermore, the integrated approach is useful for the identification of proteins in species whose genome/transcriptome is not yet annotated and characterized, so there are no sequences available on public databases. This approach relies on a custom-made putative protein database obtained by the translation of the six reading frames of each transcriptome sequence that becomes the template for the identification of proteins using experimental data derived from the proteomic analysis. In this work we built the *C. quitensis* protein database using the SEQtools software, thus obtaining a total of 993,048 predicted amino acid sequences. The peptide sequences obtained by nanoLC-MS/MS were compared with the putative amino acid sequences of the *C. quitensis* protein database allowing to identify with certainty the proteins really expressed.

### 3.2. Proteomic and Functional Enrichment Analysis

**Figure 2:**
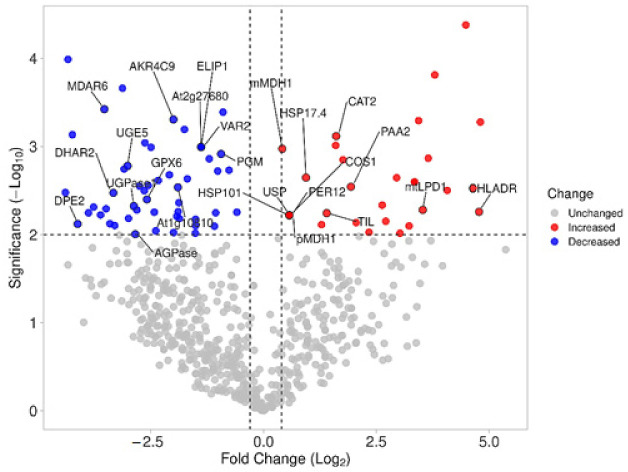
The volcano plot representation of total identified proteins in OTC and OUT samples. In red and blue are reported the statistically significant (*p*-value < 0.01) up- (log_2_FC > 0.4) and downregulated (log_2_FC < −0.5) proteins, respectively. In gray are reported the non-significant proteins (*p*-value ≥ 0.01 and/or −0.5 ≤ log_2_FC ≤ 0.4). The gene names of *A. thaliana* orthologs are reported for the representative proteins of the pathways discussed.

The effects at proteomic level of the *C. quitensis* growth inside OTC were explored in comparison to the natural growth conditions (control) by using a label free differential proteomic approach. Equal amounts of protein extracts from two biological replicates of *C. quitensis* leaves sampled inside and outside OTC were fractionated by SDS-PAGE and the 19 slices cut from each lane were in situ hydrolyzed by trypsin. Peptide mixtures for each condition were analyzed in duplicate by nanoLC-MS/MS. MSMS raw data were employed for protein identification and quantification as described in the methods section, by using MaxQuant search engine. A total of 709 proteins (excluding the 25 contaminants) were identified by employing the in-house built *Colobanthus quitensis* protein database (Table S2). This number was largely higher in comparison to the number of entries (533 excluding the 25 contaminants) obtained from a preliminary search analysis carried out by using the same raw data and search parameters for protein identification within the generalist Viridiplantae database (Table S3). This remarkable finding highlights the importance of using specific protein sequence databases for a more complete and reliable characterization of every peculiar organism. This transcriptomic-proteomic integrated approach allowed for the first time a complete molecular description of *C. quitensis* adaptation to very extreme climatic conditions. The variations of protein expression profiles were evaluated by measuring the number of spectral counts applying RSc method for their normalization and the mean between the technical replicates was calculated. Statistical analysis disclosed 93 significantly differentially expressed proteins (*p* < 0.01). By considering a log_2_ fold change cutoff between −0.3 and 0.4, 41 proteins resulted upregulated and 52 downregulated in the OTC-grown plants in comparison to the control (Figure 2).

The list of differentially expressed proteins (DEPs) is shown in Table 1 and Table 2 (the complete mass spectrometry data for each protein are reported in Table S4). The Arabidopsis Information Resource (TAIR) code and description, the *p*-value, and the log_2_ of the fold change (FC) calculated as the RSc ratio of OTC vs. OUT samples are reported for each protein. In few cases, sequences that code for different proteins hold the same TAIR description as well as sequences with the same TAIR code and TAIR description code for different isoforms (i.e., AT5G44120).

**Figure 3:**
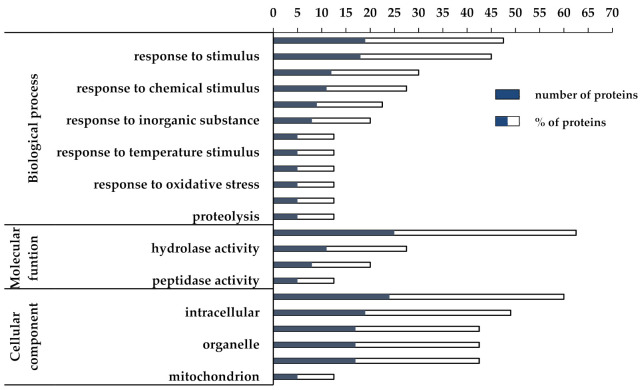
GO functional classification of the upregulated DEPs. The blue bar represents the number of upregulated proteins included in each GO term, whereas the sum of the blue and white bars represents the corresponding protein percentage.

**Figure 4:**
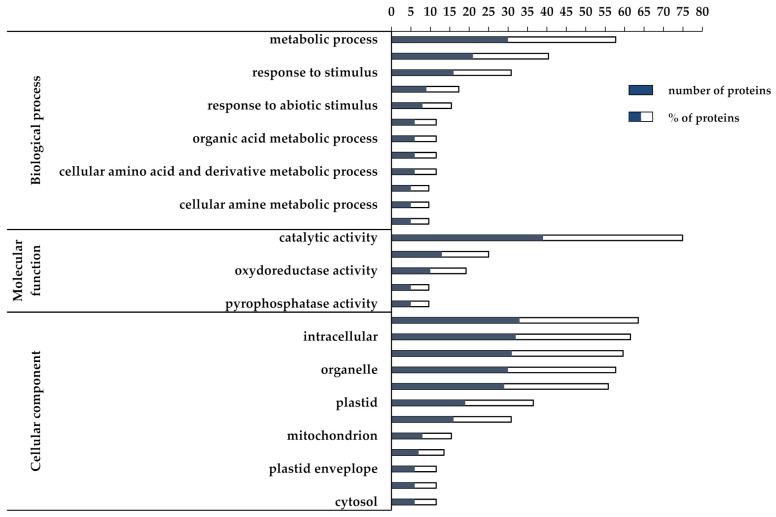
GO functional classification of the downregulated DEPs. The blue bar represents the number of downregulated proteins included in each GO term, whereas the sum of blue and white bars represents the corresponding protein percentage.

In order to achieve a fast identification of the biological relevance of DEPs, a gene ontology (GO) functional enrichment analysis was performed by means of the AgriGO toolkit (http://bioinfo.cau.edu.cn/agriGO/analysis.php, accessed on 15 May 2019) [25] using the TAIR code of orthologous Arabidopsis proteins as input. The classification of up- and downregulated DEPs into the biological process, molecular function, and cellular components domains is shown in Figure 3 and Figure 4, respectively. Regarding the upregulated DEPs, the biological process domain was found to be highly enriched in the “metabolic process” (GO:0008152, FDR < 0.05) (46%) and “response to stimulus” (GO:0050896, FDR < 0.001) (44%) terms. In particular, 19/41 upregulated DEPs were disclosed into the “metabolic process” term, which is a broad category comprising multiple molecular activities, whereas 18/41 DEPs were included in the “response to stimulus” term (GO:0050896, FDR < 0.001), encompassing “response to abiotic stimulus”, “response to stress”, and “response to chemical stimulus” terms (Figure 3).

Among the DEPs related to response to stimulus, we found proteins known to enhance plant tolerance to heat shock and oxidative stress, such as heat shock proteins (namely, HSP101 (AT1G74310) and HSP20-like (HSP17.4, AT1G54050)), temperature-induced lipocalin (TIL, AT5G58070), adenine nucleotide alpha hydrolases-like superfamily protein (universal stress protein, AT3G11930), catalase (CAT2, AT4G35090), peroxidase (AT1G71695) (Table 1). Interestingly, we also found the upregulation of the suppressor of coronatine insensitive 1 (COS1, AT2G44050) (Table 1), which plays a role in the plant response against biotic stress. Indeed, COS1 impairs Jasmonate-mediated plant response against insect and necrotrophic pathogen attacks [32]. According to this finding, it could be hypothesized that *C. quitensis* grown inside OTCs saves energy by avoiding the activation of wasteful signaling pathways, and at the same time hijacking resources towards defense against abiotic stresses. This hypothesis can be strengthened by the evidence that several DEPs within the biological process term are related to the response to different abiotic stresses and none to biotic stress (Figure 3). Besides the response to stimulus, 5/41 upregulated DEPs (12.5%) fall into the categories of “cellular catabolic process” (GO:0044248) and the same percentage was found for the “proteolysis” (GO:0006508) term.

As for the downregulated DEPs, 30/52 DEPs (58%) matched with the “metabolic process term” (GO:0008152, FDR < 0.001) and 16/52 DEPs (31%) were included in the “response to stimulus” term (GO:0050896, FDR < 0.01). The last term comprises “response to abiotic stimulus”, “response to stress”, and “response to cold” (Figure 4).

Among the latter DEPs, we found two proteins involved in photodamage recovery and photoprotection, respectively variegated 2 (VAR2, AT2G30950) and early light-inducible protein ELIP1 (AT3G22840) (Table 2), whose downregulation indicates that OTC plants are less affected by high irradiation damage than OUT plants. Conversely, this would lead to the hypothesis that photoprotection mechanisms are more efficient in OTC plants. As for the metabolic process term, most of the DEPs belonged to cellular nitrogen compound (GO:0034641) and amine (GO:0009308) metabolic process as well as cellular ketone (GO:0042180) and organic acid metabolic process (GO:0006082). Interestingly, inside these categories we found proteins involved in amino acids and complex carbohydrates biosynthesis.

At first glance, the functional enrichment analysis of up- and downregulated DEPs suggests that there is a general over-expression of proteins involved in the plant response to various abiotic stresses in OTC samples and that these plants rely on more efficient photodamage protection mechanisms. Beside this general achievement, further hypotheses have been made on how growth within OTCs can affect the physiology of *C. quitensis* plant.

### 3.3. Photorespiration Protects Photosynthetic Apparatus Counteracting Photooxidation in C. quitensis Plants under Warming Conditions

*C. quitensis* plants are highly adapted to the environmental stressors they have to face, so that even a small change in life conditions could have a strong impact on plant physiology if compared to the same change experienced in a less extreme environment [33]. Cold adaptation traits include peculiar foliar anatomy and freezing tolerance mechanisms, as well as the ability to photosynthesize under different irradiance levels and temperatures [34]. Indeed, constitutive leaf anatomical traits involved in tolerance to harsh environmental conditions (e.g., high cell wall thickness) were found to cause low mesophyll conductance whose negative effect was counteracted by the presence of the high-CO_2_ affinity Rubisco enzyme [4]. Nevertheless, simultaneous episodes of high irradiance and low temperature can cause an energy imbalance and high PSII excitation pressure which promote oxidative stress leading to photoinhibition [35]. It has been widely suggested that photorespiration may play an important role in protecting C3 plants against photooxidation, and abiotic stress in general, thus preventing an excessive production of ROS [36,37]. Indeed, the role of photorespiration has been largely re-evaluated in recent times as a key ancillary component of photosynthesis and therefore of the global carbon cycle [38]. In our atmosphere, one third of the ribulose 1,5-bisphosphate molecules become oxygenated at moderate temperature and even more in a warm environment forcing the respiratory 2-phosphoglycolate recycling that accelerates net carbon assimilation [38]. Moreover, photorespiration can help dissipating the excess of reducing equivalents and energy, thus controlling energy/redox imbalance. Photorespiratory reactions serve as direct sinks for photosynthetically generated ATP, NADPH, and reduced ferredoxin, in order to regenerate acceptors for the primary reactions [39]. Any disturbance of the balance between the supply and demand of ATP/NADPH can lead to the accumulation of ROS and consequent damage of cell components including photodamage of photosystems [40]. In this study, many enzymes supporting the photorespiratory pathway efficiency were found to be over-expressed in plants grown inside OTCs. In particular, differential proteomic analysis revealed the over-expression of both mitochondrial (mMDH1, AT1G53240) and peroxisomal malate dehydrogenase (PMDH1, AT2G22780) in OTC samples (log_2_FC = 0.41 and 0.57, respectively) (Table 1). MDHs are the key enzymes of the malate valves which use malate/oxaloacetate (OAA) shuttles to balance the ATP/NAD(P)H ratio between cellular compartments [41]. Mitochondrial MDH is reported to play an important role in photorespiration, being involved in lowering the concentration of NADH equivalents produced during the oxidation of glycine to serine by the glycine decarboxylase complex (GDC), thus preventing its inhibitory effect on the enzyme activity [42]. Accordingly, a *mmdh1mmdh2* Arabidopsis mutant, lacking mMDH activity, showed altered photorespiration parameters and higher Gly levels than the wild type, due to the imbalance of glycine decarboxylation reaction. Moreover, it showed an enhanced leaf respiration suggesting that mMDH normally uses NADH to reduce oxaloacetate to malate, rather than to drive mitochondrial respiration [43]. The recovery of mMDH activity led to suppression of respiratory rate, improvement of photorespiration, and an increase of plant growth, suggesting a role of mMDH in these processes [43]. The requirement of NADH for hydroxypyruvate reduction in peroxisomes would provide an additional sink for reducing equivalents, and peroxisomal MDH is involved in the conversion of malate to OAA to guarantee the optimal rate of photorespiration [44]. The over-expression of this enzyme in OTC samples supports the hypothesis that plants grown in warmer conditions display higher photorespiration rates. Interestingly, the mitochondrial dihydrolipoyl dehydrogenase (mtLPD1, AT1G48030) was also found to be strongly over-expressed in OTC samples (log_2_FC = 3.53) (Table 1). Mitochondrial LPD is a crucial component of important metabolic protein complexes, such as pyruvate dehydrogenase, 2-oxoglutarate dehydrogenase, branched-chain 2-oxoacid dehydrogenase, and glycine decarboxylase complexes. Arabidopsis plants over-expressing mtLPD were reported to have increased photorespiration and CO_2_ assimilation rates as well as enhanced plant growth [45]. In particular, the level of several diagnostic metabolites of TCA cycle and photorespiration were found significantly decreased in the over-expression lines, suggesting that elevated mtLPD activity might facilitate carbon flow through these pathways, thus lowering the accumulation of photorespiratory metabolites which impair both Rubisco and other Calvin–Benson cycle enzymes activity. As a consequence, over-expression lines showed enhanced CO_2_ fixation and improved light capture and light use efficiency due to a greater availability of NADP^+^ as electron acceptor at PSI [45]. Interestingly, by using the freely available STRING program, both mitochondrial and peroxisomal MDH1 as well as mtLDP1 were predicted to interact each other confirming their involvement in a network of physiological processes strictly connected to *C. quitensis* response to warming (Figure S2A). Thus, we can hypothesize that under warming conditions, mtLPD, together with mMDH and PMDH, may enhance photorespiration efficiency in *C. quitensis*, thus controlling the energy/redox balance and stimulating photosynthetic CO_2_ assimilation with positive effect on plant growth and biomass production. Although photorespiration has often been considered a wasteful pathway associated with reduced carbon gain in C_3_ plants [39,46], recent studies revealed a positive correlation between photorespiration and yields of some highly productive wheat genotypes [47,48]. In fact, photorespiration is emerging as a crucial component of processes involved in the reduction of ROS production, protection against photoinhibition, optimization of photosynthesis and support for plant growth ([37] and references therein). Moreover, the beneficial role of photorespiration is also related to the release of intermediate molecules which can be hijacked towards other pathways. For example, H_2_O_2_ released during photorespiratory metabolism is probably involved in signal transduction that modulates plant growth and development, as well as abiotic stress responses. In addition, glycine, generated during photorespiration, can be used as a substrate for biosynthesis of glutathione, a key component of antioxidant defense systems, and both glycine and serine can also be withdrawn from the photorespiratory pathway for export [39,49].

### 3.4. Warming Conditions Influence High Irradiance-Induced Photodamage in C. quitensis

It has been reported that *C. quitensis* dissipates the excess of absorbed energy through non-photochemical quenching (NPQ), downregulating its electron transport rate and thereby minimizing oxygen reduction and the generation of reactive oxygen species [50]. Interestingly, cold-acclimated *C. quitensis* plants were found to recover from cold-induced photoinhibition better and faster than non-acclimated plants implying an intrinsic ability of Antarctic plants to overcome stressors they struggle with [51]. In this study, differential proteomic analysis revealed the under-expression of proteins involved in photoprotection and photodamage recovery, leading to the hypothesis that warming might stimulate metabolic reprogramming which protects plants against photoinhibition. In this context, the enhancement of photorespiration, experienced by *C. quitensis* plants grown inside OTCs, could play an important role in photoprotection, as reported for many plant species [52,53,54].

**Figure 5:**
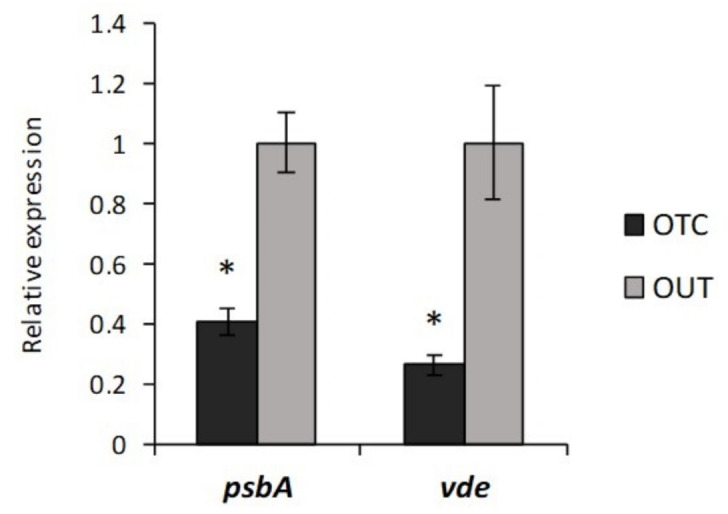
Relative expression of *C. quitensis* genes normalized with *G6PDH* and *PP2Acs* genes. *psbA*: PSII protein D1; *vde*: violaxanthin de-epoxidase. Data represent the mean ± SD from three biological and three technical replicates. Asterisks indicate significant differences (Student *t*-test * *p* ≤ 0.05).

In particular, differential proteomic analysis revealed the under-expression of VARIEGATED 2 (VAR2, AT2G30950) protein in OTC samples (log_2_FC = −1.38) (Table 2). VAR2 is an ATP-dependent metalloprotease belonging to the FtsH (Filamentation temperature sensitive) family, found in eubacteria, animals, and plants [55]. In *Arabidopsis thaliana*, 12 FtsH family members are present (FtsH1–12) and their subcellular localization is restricted to mitochondria and chloroplasts [56]. Arabidopsis knock-out mutants of *ftsh2* (*var2*) were found to be highly sensitive to photosystem II (PSII) photodamage [57] and a complex consisting of FtsH2 and FtsH5 appeared to be involved in the degradation of the PSII reaction center protein D1 which is a key process in the repair cycle of photodamaged PSII [58]. Thus, these findings indicate VAR2 as a key enzyme in plant photoprotection mechanisms. This statement was strengthened by the evidence that its expression was upregulated by high light in Arabidopsis [59] as well as in the unicellular green alga *Chlamydomonas reinhardtii* [60]. According to VAR2 under-expression, we also found that the gene coding PSII protein D1 (*psbA*) was significantly under-expressed in OTC samples (Figure 5), highlighting a reduced turnover rate of this protein. Interestingly, in this work we also disclosed the under-expression of ELIP1 (early light-inducible protein 1, AT3G22840) in OTC samples (log_2_FC = −1.38) (Table 2). ELIPs belong to a class of proteins structurally related to the chlorophyll- and carotenoid-binding light harvesting complexes (LHCs) and accumulate transiently under conditions of high light intensities when the expression of LHC is downregulated [61,62]. This protein family has been hypothesized to play a role in photoprotection either by maintaining a low level of free chlorophyll, thus avoiding ROS formation, or by promoting zeaxanthin-dependent photoprotection [63,64]. A green algal homologue of ELIPs (Cbr), upregulated under light stress, was supposed to bind zeaxanthin and have a role in zeaxanthin-associated photoprotection [65,66]. According to ELIP1 under-expression, we also found the downregulation of the gene coding the xanthophyll cycle enzyme violaxanthin de-epoxidase (VDE) (Figure 5), which contributes to the dissipation of excess light energy as heat through NPQ mechanism by transforming violaxanthin to zeaxanthin in high light [67]. This result strengthens the hypothesis that *C. quitensis* plants grown under warming conditions could be less exposed to photooxidative damage than the counterpart grown in open field.

### 3.5. Temperature-Induced Lipocalin (TIL) and HSPs Contribute to Oxidative-Stress Control

Lipocalins are a group of widely distributed proteins that have been found in bacteria, invertebrates, vertebrates and plants [68]. Plants’ lipocalins are classified as temperature-induced lipocalin (TIL) and chloroplastic lipocalin (CHL) [69]. Recently, Boca and colleagues [70] demonstrated overlapping functions between AtCHL and AtTIL in Arabidopsis leaves and the involvement of both lipocalins in oxidative stress tolerance. In particular, AtTIL, as a peripheral membrane protein, was reported to alleviate heat-induced oxidative stress on Arabidopsis membrane limiting the accumulation of lipid peroxidation products [71]. Interestingly, in this work the TIL protein (AT5G58070) was found to be over-expressed in OTC samples (log_2_FC = 1.4) (Table 1), thus we assumed its involvement in the oxidative-stress control. Moreover, differential proteomic analysis also revealed the over-expression of some heat shock proteins (HSPs), widely recognized as molecular effectors for oxidative stress protection [72,73]. In particular, HSP101 (AT1G74310), belonging to the Hsp100/ClpB family, and HSP17.4 (AT1G54050), belonging to the HSP20-like family, showed a log_2_FC equal to 0.57 and 0.94, respectively (Table 1). HSPs were first identified as strongly induced by heat stress signaling molecules, such as plant hormones and ROS, and are now known to be expressed in response to a wide range of other environmental threats, such as drought, salinity, cold and oxidative stress [72,74]. They function as molecular chaperones contributing to cellular homeostasis under optimal and sub-optimal growth conditions, and play complementary roles in protecting proteins against stress [72]. In particular, their ability to maintain glutathione in its reduced form, even under oxidative conditions, is driving the emerging evidence that these proteins are capable of decreasing the intracellular level of ROS in a glutathione-dependent way, restoring redox homeostasis [73]. Moreover, many other proteins cooperate to control oxidative stress in OTC samples, such as the peroxisomal catalase CAT2 (AT4G35090, log_2_FC = 1.61), the peroxidase PER12 (AT1G71695, log_2_FC = 0.57) and the chloroplast aldehyde reductase ChlADR (NAD(P)-binding Rossmann-fold superfamily protein, AT1G54870, log_2_FC = 4.78) (Table 1), which helps maintain the photosynthetic process by detoxifying reactive carbonyls formed during lipid peroxidation. On the other hand, we also found the downregulation of some other aldo-keto reductase isoforms (i.e., NAD(P)-linked oxidoreductase superfamily protein AT1G10810, log_2_FC = −1.90; AT2G27680, log_2_FC = −1.38; AT2G37770, log_2_FC = −1.99) (Table 2) leading to the hypothesis that plants drive the expression of specific isoforms depending on the substrate specificity and cellular localization. Moreover, two key enzymes of the ascorbate-glutathione cycle, i.e., the monodehydroascorbate reductase 6, MDAR6 (AT1G63940, log_2_FC = −3.52) and the dehydroascorbate reductase 2, DHAR2 (AT1G75270, log_2_FC = −3.32) were found to be downregulated (Table 2). These enzymes are believed to protect plants from oxidative stress by recycling the antioxidant ascorbic acid. Arabidopsis contains six MDAR isoforms, and three DHAR isoforms, with various cellular localization and different contribution to ascorbate recycling [75,76]. Specifically, MDAR6 is targeted to mitochondria or plastids [75], while DHAR2 is localized in the cytosol [76]. Recent studies demonstrated that independent Arabidopsis *dhar2* and *mdar6* mutants were not strongly affected on the levels and redox state of ascorbate implying a small contribution of these isoforms to the ascorbate recycling [77,78]. It is worthwhile considering that *C. quitensis* plants have been shown to activate many systems leading to oxidative-stress control as well as H_2_O_2_ reducing reactions that take place independently from ascorbate oxidation. Moreover, the depletion of reduced glutathione by active DHAR, which uses GSH to reduce dehydroascorbate to ascorbate, could impair the activity of HSPs which need GSH to maintain proteins in their functional conformations. This hypothesis is also in line with the observed under-expression of the glutathione peroxidase 6, GPX6 (AT4G11600, log_2_FC = −2.58). Interestingly, DHAR2 was found to interact with both MDAR6 and GPX6 using STRING program (Figure S2B).

Overall, we can infer that OTC plants are able to control oxidative stress due to the over-expression of some antioxidant enzymes and a series of temperature-induced proteins known to be involved in maintaining the redox state. The concomitant under-expression of known ROS scavengers can be justified by the need to minimize energy expenditure by channeling energy only on the expression of the isoforms deemed necessary on the basis of the relative substrates and the specific cellular localization.

### 3.6. Antioxidant Enzyme Activity Assays

In order to confirm the hypothesis that OTC plants are able to control oxidative stress damage in a more powerful way than OUT plants, we compared the activity of some antioxidant enzymes in both samples.

**Figure 6:**
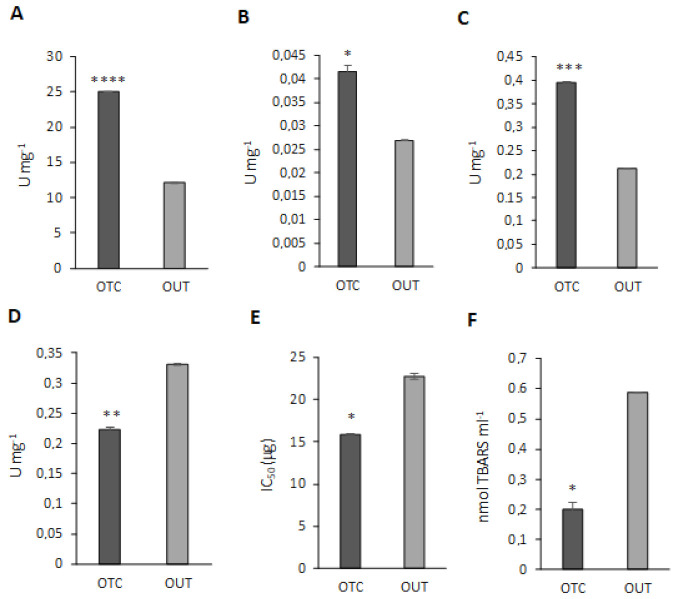
Antioxidant enzyme activities and TBARS content in *C. quitensis* leaves. (**A**) Catalase (CAT); (**B**) glutathione S-transferase (GST); (**C**) guaiacol peroxidase (POD); (**D**) ascorbate peroxidase (APX); (**E**) superoxide dismutase (SOD); (**F**) TBARS content. Data represent the mean ± SD from three biological and three technical replicates. Asterisks indicate significant differences (Student *t*-test: * *p* < 0.05; ** *p* < 0.01; *** *p* < 0.001; **** *p* < 0.00001).

As shown in Figure 6, the activities of catalase (CAT), glutathione S-transferase (GST), guaiacol peroxidase (POD) and superoxide dismutase (SOD) were all significantly higher in OTC samples, which also showed a significantly lower TBARS content entailing a better restraint of lipid peroxidation events. As for SOD, it is worthwhile remembering that its activity was expressed as IC_50_, corresponding to the amount (µg) of total protein extract necessary to inhibit by 50% the reduction of a tetrazolium salt by the superoxide anion. Thus, low levels of IC_50_ are related to higher SOD activity. On the other hand, ascorbate peroxidase activity was found to be significantly lower in OTC than OUT plants. Peroxidases are enzymes involved in maintaining H_2_O_2_ homeostasis, together with peroxisomal catalase. In particular, ascorbate peroxidases participate in the removal of H_2_O_2_ as part of the ascorbate-glutathione pathway. Indeed, in this work we found the downregulation of two other enzymes involved in the ascorbate-glutathione pathway, namely MDAR and DHAR, leading to the hypothesis that the whole pathway could be limited in OTC plants. Nevertheless, the over-expression of other enzymes, such as POD, can contribute to the removal of H_2_O_2_ in plants and compensate for the downregulation of APX. In this regard, it has been reported that rice mutants for cytosolic APXs were able to upregulate other peroxidases and to cope with abiotic stress as non-transformed plants [79]. Moreover, we could also infer that OTC plants regulate the electron transport pathway in a way that avoids the reduction of oxygen, thus limiting ROS and H_2_O_2_ production. In general, our differential proteomic analysis revealed that OTC plants activate many different routes aimed to dissipate the excess of reducing equivalents and avoid ROS production and the over-expression of ROS detoxifying enzymes is a supplementary way to maintain oxidative stress under strict control.

### 3.7. Warming Influences Carbohydrates Accumulation in C. quitensis

Carbohydrates play a key role in plant resistance to abiotic stress as they act as osmolytes, maintaining the cell–water balance and membrane stability, antioxidants, and thermoprotectants [80]. Moreover, cell wall polysaccharides provide mechanical integrity to each cell and mediate its interaction either with neighboring cells or the environment [81]. In *C. quitensis* plants, the sugar content was proved to be strongly affected by abiotic stresses, such as thermal regimen and day length [82], and sucrose concentration in leaves was positively correlated with freezing avoidance [83]. Furthermore, *C. quitensis* growth inside OTCs was found to reduce the content of cell wall chemical components (cellulose, hemicellulose and lignin), likely allowing a better CO_2_ diffusion into the leaf mesophyll [12]. Interestingly, in our work, the differential proteomic analysis revealed the under-expression of many enzymes involved in carbohydrates metabolism such as UDP-glucose pyrophosphorylase 1 (UGPase1, AT3G03250, log_2_FC = −2.87), ADP-glucose pyrophosphorylase (AGPase, AT1G74910, log_2_FC = −2.84), phosphoglucomutase (AT1G70730, log_2_FC = −0.94), disproportionating enzyme 2 (DPE2, AT2G40840, log_2_FC = −4.11), and UDP glucose/galactose epimerase 5 (UGE5, log_2_FC = −3.01). In particular, UGPase 1 is capable of activating glucose-1-P (Glc-1P) to UDP-glucose (UDP-Glc) that can be used for sucrose as well as cell wall polysaccharides biosynthesis; AGPase plays a role in the starch biosynthesis catalyzing the synthesis of ADP-Glc from Glc-1P; phosphoglucomutase drives the synthesis of Glc-1P from Glc-6P which can be utilized in both the above pathways; DPE2 catalyzes the cytosolic conversion of maltose into glucose, which can be used for sucrose biosynthesis; and UGE5 appears to be mainly involved in the generation of UDP-Galactose from UDP-Glc to generate building blocks for cell wall carbohydrates biosynthesis [84]. Furthermore, in Arabidopsis, the isoform UGE5 was also found to be induced by abscisic acid, salt, and temperature stress, and was hypothesized to act in stress situations by supplying UDP-galactose for the biosynthesis of the osmoprotectant galactinol [84]. All in all, these findings reveal that warming slows down carbohydrate metabolism in *C. quitensis* plants and this event may impair plant freezing resistance, due to the important role of sugar metabolites as cryo-protectants and as adaptive biochemical factors in osmo-protection during abiotic stress. Indeed, it has been recently demonstrated that warmer conditions enhance the freezing vulnerability of both Antarctic species increasing their LT_50_ (freezing temperature producing 50% photoinactivation) [13]. Similar responses were also reported for temperate and arctic plants where warming altered freezing tolerance and influenced plant physiology, including carbon metabolism [85]. In addition, our results are consistent with that already reported [12] who highlighted reduced fiber content in the cell wall of *C. quitensis* grown inside OTCs. In this regard, it is worthwhile mentioning that in this work we also found the downregulation of the cynnamil-alcohol dehydrogenase CAD1 (AT1G72680, log_2_FC = −2.64), involved in lignin biosynthesis, strengthening the evidence that warming reduces the deposition of cell wall components.

## 4. Conclusions

**Figure 7:**
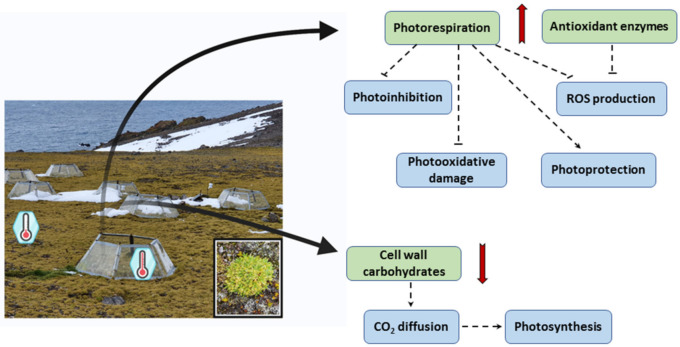
Outline of the main results obtained in the present paper. Continuous red arrows: obtained results; dashed black arrows: inferred information. The figure was created with BioRender.com, accessed on 20 April 2021.

In conclusion, our analysis revealed that OTCs plants have a high rate of photorespiration which acts as a protective mechanism against photooxidative damage and ROS production (Figure 7). Moreover, *C. quitensis* plants grown inside OTCs seemed to control the oxidative stress more efficiently than the counterpart grown in open field by means of scavengers which detoxify ROS and limit lipid peroxidation. Furthermore, the carbohydrates metabolism reprogramming could lead to reducing the deposition of cell wall polysaccharides, probably improving CO_2_ diffusion into the mesophyll and therefore the photosynthetic efficiency. Hence, it seems that OTCs growth is beneficial for *C. quitensis* plants, which come closer to their optimal temperature for net photosynthesis, although the drawback is a reduced freezing tolerance due to lower concentration of soluble sugars which act as osmoprotectants.

## Figures and Tables

**Table 1:** List of the 41 upregulated proteins.

TAIR Code	TAIR Description	*p*-Value	log_2_FC OTC/OUT
AT2G28490	RmlC-like cupins superfamily protein	5.29 × 10^−4^	4.81
AT1G54870	NAD(P)-binding Rossmann-fold superfamily protein	5.49 × 10^−3^	4.78
AT5G60160	Zn-dependent exopeptidases superfamily protein	2.95 × 10^−3^	4.64
AT5G44120	ATCRA1, CRA1, CRU1, RmlC-like cupins superfamily protein	4.18 × 10^−5^	4.48
AT5G44120	ATCRA1, CRA1, CRU1, RmlC-like cupins superfamily protein	3.14 × 10^−3^	4.07
AT3G54470	Uridine 5’-monophosphate synthase/UMP synthase (PYRE-F) (UMPS)	1.54 × 10^−4^	3.80
AT3G51640	Stress response NST1-like protein	1.36 × 10^−3^	3.65
AT1G48030	mtLPD1, mitochondrial lipoamide dehydrogenase 1	5.23 × 10^−3^	3.53
AT1G04580	AAO4, AO4, ATAO-4, ATAO2, aldehyde oxidase 4	5.10 × 10^−4^	3.44
AT1G79530	GAPCP-1, glyceraldehyde-3-phosphate dehydrogenase of plastid 1	2.51 × 10^−3^	3.35
AT2G15220	Plant basic secretory protein (BSP) family protein	2.51 × 10^−3^	3.35
AT3G22640	PAP85, cupin family protein	7.96 × 10^−3^	3.23
AT2G24530	Transcriptional regulator of RNA poIII, SAGA	9.63 × 10^−3^	3.03
AT3G09540	Pectin lyase-like superfamily protein	2.25 × 10^−3^	2.95
AT1G43670	Inositol monophosphatase family protein (CYFBP, Fructose-1,6-bisphosphatase, cytosolic)	7.04 × 10^−3^	2.71
AT4G25150	HAD superfamily, subfamily IIIB acid phosphatase	4.62 × 10^−3^	2.63
AT5G19660	ATS1P, ATSBT6.1, S1P, SITE-1 protease	9.39 × 10^−3^	2.34
AT1G13750	Purple acid phosphatases superfamily protein	7.29 × 10^−3^	2.05
AT2G05840	PAA2, 20S proteasome subunit PAA2	2.86 × 10^−3^	1.93
AT5G67360	ARA12, subtilase family protein	1.42 × 10^−3^	1.76
AT4G35090	CAT2, catalase 2	7.64 × 10^−4^	1.61
AT2G24200	Cytosol aminopeptidase family protein (ATLAP1, LAP1, LEUCYL AMINOPEPTIDASE 1)	9.74 × 10^−4^	1.60
AT5G58070	ATTIL, TIL, temperature-induced lipocalin	5.70 × 10^−3^	1.40
AT1G03090	MCCA, methylcrotonyl-CoA carboxylase alpha chain, mitochondrial/3-methylcrotonyl-CoA carboxylase 1 (MCCA)	7.70 × 10^−3^	1.28
AT1G54050	HSP20-like chaperones superfamily protein	2.25 × 10^−3^	0.94
AT2G22780	PMDH1, peroxisomal NAD-malate dehydrogenase 1	6.05 × 10^−3^	0.57
AT1G31190	IMPL1, myo-inositol monophosphatase like 1	5.98 × 10^−3^	0.57
AT1G59730	ATH7, TH7, thioredoxin H-type 7	5.98 × 10^−3^	0.57
AT1G71695	Peroxidase superfamily protein	5.98 × 10^−3^	0.57
AT1G74310	ATHSP101, HOT1, HSP101, heat shock protein 101	5.98 × 10^−3^	0.57
AT2G05030	Transposable element gene	5.98 × 10^−3^	0.57
AT2G05710	ACO3, aconitase 3	5.98 × 10^−3^	0.57
AT2G25890	Oleosin family protein	5.98 × 10^−3^	0.57
AT2G29570	ATPCNA2, PCNA2, proliferating cell nuclear antigen 2	5.98 × 10^−3^	0.57
AT2G44050	COS1, COS1, 6,7-dimethyl-8-ribityllumazine synthase/DMRL synthase/lumazine synthase/riboflavin synthase	5.98 × 10^−3^	0.57
AT3G01570	Oleosin family protein	5.98 × 10^−3^	0.57
AT3G11930	Adenine nucleotide alpha hydrolases-like superfamily protein	5.98 × 10^−3^	0.57
AT3G23490	CYN, cyanase	5.98 × 10^−3^	0.57
AT3G55620	emb1624, translation initiation factor IF6	5.98 × 10^−3^	0.57
AT4G16260	Glycosyl hydrolase superfamily protein	5.98 × 10^−3^	0.57
AT1G53240	mMDH1, lactate/malate dehydrogenase family protein	1.06 × 10^−3^	0.41

**Table 2:** List of the 52 downregulated proteins.

TAIR Code	TAIR Description	*p*-Value	log_2_FC OTC/OUT
AT5G05200	Protein kinase superfamily protein	3.33 × 10^−3^	−4.39
AT1G77510	RPT3, regulatory particle triple-A ATPase 3	1.03 × 10^−4^	−4.33
AT5G58290	RPT3, regulatory particle triple-A ATPase 3	7.35 × 10^−4^	−4.23
AT2G40840	DPE2, disproportionating enzyme 2	7.56 × 10^−3^	−4.11
AT2G20420	ATP citrate lyase (ACL) family protein	5.65 × 10^−3^	−3.88
AT3G23570	Alpha/beta-Hydrolases superfamily protein	4.85 × 10^−3^	−3.76
AT1G79870	D-isomer specific 2-hydroxyacid dehydrogenase family protein	5.97 × 10^−3^	−3.61
AT1G63940	MDAR6, monodehydroascorbate reductase 6	3.78 × 10^−4^	−3.52
AT5G17380	Thiamine pyrophosphate dependent pyruvate decarboxylase family protein	5.08 × 10^−3^	−3.49
AT1G11860	Glycine cleavage T-protein family	7.50 × 10^−3^	−3.40
AT1G75270	DHAR2, dehydroascorbate reductase 2	3.35 × 10^−3^	−3.32
AT5G48540	Receptor-like protein kinase-related family protein	7.88 × 10^−3^	−3.30
AT3G55270	ATMKP1, MKP1, mitogen-activated protein kinase phosphatase 1	2.18 × 10^−4^	−3.12
AT4G18810	NAD(P)-binding Rossmann-fold superfamily protein	1.80 × 10^−3^	−3.09
AT4G10960	UGE5, UDP-D-glucose/UDP-D-galactose 4-epimerase 5	1.66 × 10^−3^	−3.01
AT4G11150	emb2448, TUF, TUFF, VHA-E1, vacuolar ATP synthase subunit E1	6.54 × 10^−3^	−2.99
AT3G03250	AtUGP1, UGP, UGP1, UDP-GLUCOSE PYROPHOSPHORYLASE 1	4.73 × 10^−3^	−2.87
AT1G74910	ADP-glucose pyrophosphorylase family protein	9.88 × 10^−3^	−2.84
AT1G48830	Ribosomal protein S7e family protein	5.25 × 10^−3^	−2.81
AT1G53450	Epstein-barr nuclear antigen	2.81 × 10^−3^	−2.74
AT1G72680	ATCAD1, CAD1, cinnamyl-alcohol dehydrogenase	3.17 × 10^−3^	−2.64
AT5G26780	SHM2, serine hydroxymethyltransferase 2	9.12 × 10^−4^	−2.62
AT4G11600	ATGPX6, GPX6, LSC803, PHGPX, glutathione peroxidase 6	3.98 × 10^−3^	−2.58
AT2G45300	RNA 3’-terminal phosphate cyclase/enolpyruvate transferase, alpha/beta	2.76 × 10^−3^	−2.55
ATCG01280	YCF2.2, chloroplast Ycf2;ATPase, AAA type, core	1.03 × 10^−3^	−2.49
AT1G19920	APS2, ASA1, pseudouridine synthase/archaeosine transglycosylase-like family protein	5.55 × 10^−3^	−2.42
AT1G09310	Protein of unknown function, DUF538	9.05 × 10^−3^	−2.39
AT5G46290	KAS I, KAS1, 3-ketoacyl-acyl carrier protein synthase I	2.42 × 10^−3^	−2.33
AT3G53580	Diaminopimelate epimerase family protein	2.09 × 10^−3^	−2.08
AT2G30110	ATUBA1, MOS5, UBA1, ubiquitin-activating enzyme 1	9.46 × 10^−3^	−1.99
AT2G37770	NAD(P)-linked oxidoreductase superfamily protein (AKR4C9, ALDO-KETO REDUCTASE FAMILY 4 MEMBER C9, CHLAKR, CHLOROPLASTIC ALDO-KETO REDUCTASE)	4.95 × 10^−4^	−1.99
AT5G17770	ATCBR, CBR, CBR1, NADH:cytochrome B5 reductase 1	6.18 × 10^−3^	−1.92
AT1G10810	NAD(P)-linked oxidoreductase superfamily protein	2.89 × 10^−3^	−1.90
AT1G09130	ATP-dependent caseinolytic (Clp) protease/crotonase family protein	5.48 × 10^−3^	−1.89
AT5G61510	GroES-like zinc-binding alcohol dehydrogenase family protein	4.33 × 10^−3^	−1.87
AT2G42590	GF14 MU, GRF9, general regulatory factor 9	6.54 × 10^−3^	−1.86
AT3G49830	P-loop containing nucleoside triphosphate hydrolases superfamily protein	6.41 × 10^−4^	−1.75
AT2G22910	NAGS1, N-acetyl-l-glutamate synthase 1	2.32 × 10^−3^	−1.69
AT4G17170	AT-RAB2, ATRAB-B1B, ATRAB2A, ATRABB1C, RAB-B1B, RAB2A, RABB1C, RAB GTPase homolog B1C	9.62 × 10^−3^	−1.51
AT2G22780	PMDH1, peroxisomal NAD-malate dehydrogenase 1	6.68 × 10^−3^	−1.50
AT3G51800	ATEBP1, ATG2, EBP1, metallopeptidase M24 family protein	7.91 × 10^−3^	−1.50
AT2G27680	NAD(P)-linked oxidoreductase superfamily protein	1.01 × 10^−3^	−1.38
AT2G30950	FTSH2, VAR2, FtsH extracellular protease family	1.02 × 10^−3^	−1.38
AT3G22840	ELIP, ELIP1, chlorophyll A-B binding family protein	1.02 × 10^−3^	−1.38
AT1G62780	Dimethylallyl, adenosine tRNA methylthiotransferase	1.38 × 10^−3^	−1.20
AT4G24280	cpHsc70-1, chloroplast heat shock protein 70-1	8.01 × 10^−3^	−1.09
AT2G21530	SMAD/FHA domain-containing protein	5.64 × 10^−3^	−1.06
AT5G17920	ATCIMS, ATMETS, ATMS1, cobalamin-independent synthase family protein	1.89 × 10^−3^	−1.01
AT1G70730	Phosphoglucomutase/phosphomannomutase family protein (PGM)	1.22 × 10^−3^	−0.94
AT3G04770	RPSAb, 40s ribosomal protein SA B	4.08 × 10^−4^	−0.90
AT4G35090	CAT2, catalase 2	1.85 × 10^−3^	−0.76
AT1G80380	P-loop containing nucleoside triphosphate hydrolases superfamily protein	5.56 × 10^−3^	−0.59

## Data Availability

LC-MS/MS raw data are available in the ProteomeXchange repository, using the following identifiers: Project Name: Colobanthus quitensis differential proteomics; Project accession: PXD027038.

## Supplementary Materials

The following are available online at https://www.mdpi.com/article/10.3390/biom11081094/s1, Figure S1: Melting curves of *psbA* (PSII protein D1) and *vde* (violaxanthin de-epoxidase) genes, Figure S2: The protein–protein interaction network of differentially expressed proteins (DEPs) in OTC vs. OUT *C. quitensis* leaf samples, Table S1: List of primers used in the study and related information, Table S2: Complete protein table obtained by MaxQuant using the in-house built *C. quitensis* protein database, Table S3: Complete protein table obtained by MaxQuant using the Viridiplantae database, Table S4: List of the differentially expressed proteins.
