# Supplementary material for: What Antarctic Plants Can Tell Us about Climate Changes: Temperature as a Driver for Metabolic Reprogramming
Source: Biomolecules. 2021 Jul 23;11(8):1094. doi: 10.3390/biom11081094 (PMC8392395; doi:10.3390/biom11081094)
Supplement: Supplementary file 1 [file biomolecules-11-01094-s001.zip › Table S1.pdf]

Table S1. List of primers used in the study and related information.

Gene	Gene description	Primer sequences (5′-3′) forward/reverse	Annealing Temperature (°C)	Primers concentration (nM)	Amplicon length (bp)	Efficiency (*E*) (%)	R^2
psbA	Photosystem II protein D1	TGGTGGAGCAGCGATGAAAG<br>GGGAACGTTTCTGCTCTTGGA	60	200	131	93.5	0.990
vde	Violaxanthin de-epoxidase	CGTCTGCACAAGCTGTTGAT<br>ATCTGGCACTCCGTTTCATC	64	250	157	106.0	0.995
