# Supplementary figures and images for: What Antarctic Plants Can Tell Us about Climate Changes: Temperature as a Driver for Metabolic Reprogramming
Source: Biomolecules. 2021 Jul 23;11(8):1094. doi: 10.3390/biom11081094 (PMC8392395; doi:10.3390/biom11081094)

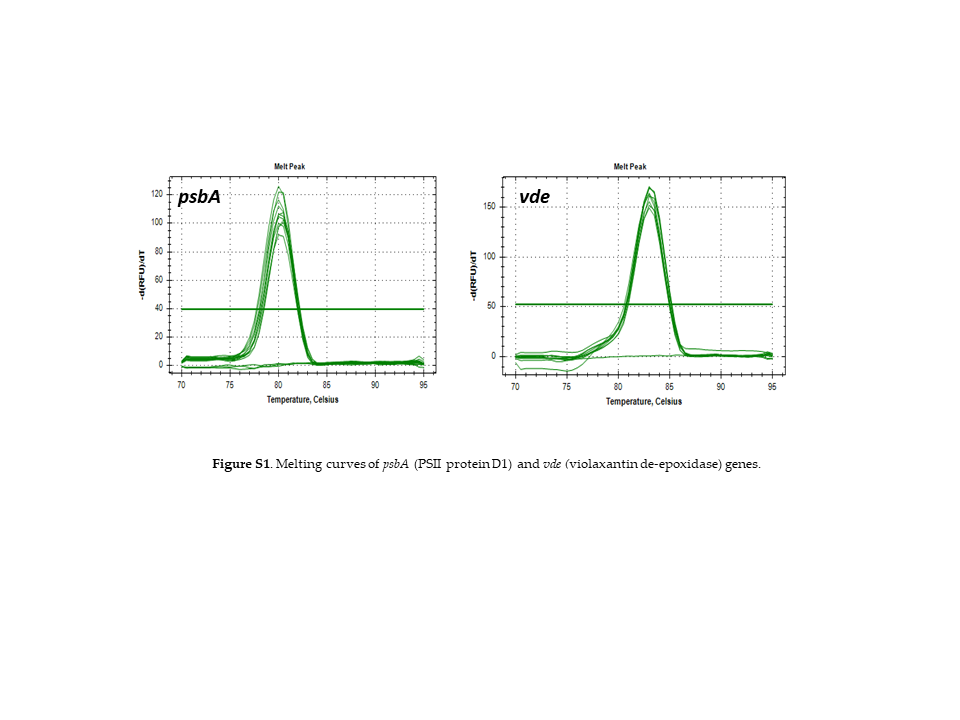

Supplement: Supplementary file 1 [file biomolecules-11-01094-s001.zip › Figure S1.png]

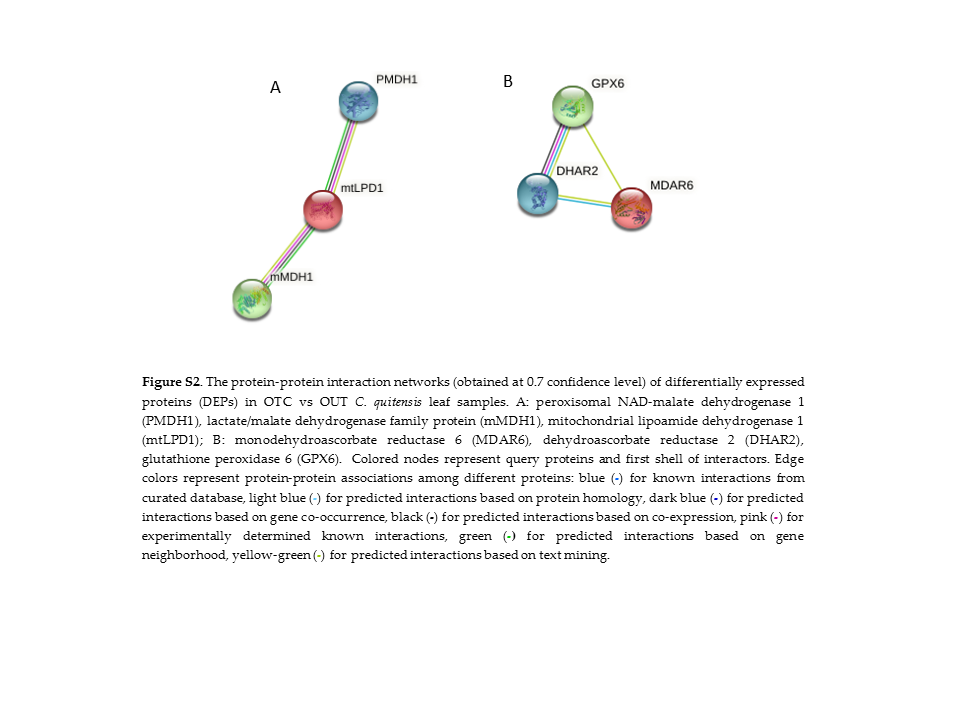

Supplement: Supplementary file 1 [file biomolecules-11-01094-s001.zip › Figure S2.png]
